# Smoking Habits and Attitudes toward Smoking in Patients with Severe Mental Illness in Residential Facilities in Insular Greece

**DOI:** 10.3390/healthcare11050642

**Published:** 2023-02-21

**Authors:** Ioanna Botsari, Georgia Marouli, Aikaterini Arvanitaki, Vaios Peritogiannis

**Affiliations:** 1 “Skitali” Day Center, Society for the Promotion of Mental Health in Epirus, 45445 Ioannina, Greece; 2 “Agia Eirini” General Hospital of Corfu, 49100 Corfu, Greece; 3 Mobile Mental Health Unit of the Prefectures of Ioannina and Thesprotia, Society for the Promotion of Mental Health in Epirus, 45445 Ioannina, Greece

**Keywords:** attitudes and habits, mental healthcare staff, residential rehabilitation facilities, schizophrenia, severe mental illness, smoking

## Abstract

Smoking may contribute to increased cardiovascular morbidity and mortality in patients with schizophrenia spectrum disorders. The objective of the present study is to explore the attitudes toward smoking in patients with severe mental illness in residential rehabilitation facilities in insular Greece. The patients (n = 103) were studied with the use of a questionnaire based on a semi-structured interview. Most of the participants (68.3%) were current regular smokers, had been smoking for 29 years and started smoking at an early age. The majority (64.8%) reported having tried to quit smoking in the past, and only half had been advised by a physician to quit. The patients agreed on the rules for smoking and believed that the staff should avoid smoking in the facility. The years of smoking were statistically significantly correlated to the educational level and the treatment with antidepressant medication. A statistical analysis showed that longer stay period in the facilities correlates with current smoking, an effort to quit and increased belief that smoking causes harm to health. Further research on the attitudes of patients in residential facilities toward smoking is needed, which could guide interventions for smoking cessation and should be assumed by all health professionals who are involved in the care of those patients.

## 1. Introduction

Schizophrenia and related syndromes are still the most devastating severe mental illnesses (SMI), according to the most recent Global Burden of Disease Study [1]. The course of schizophrenia is chronic, the prognosis is generally modest, and the long-term outcome is often poor, with high rates of disability and poor functioning [2]. Notably, life expectancy in patients with schizophrenia spectrum disorders has been reported to be 15–20 years shorter than the general population, and the gap is increasing over the decades, despite the substantial progress in the treatment of these disorders [3,4]. Despite concerns regarding the relatively high suicide rates in schizophrenia, most deaths are attributable to preventable diseases, such as cardiovascular events [5]. Cardiovascular morbidity and mortality are highly prevalent in SMI and are the result of a complex interplay of factors that act synergistically [6,7]. Lifestyle factors are widely recognized as playing an important role in the physical morbidity of patients. These include a sedentary lifestyle and poor physical activity, poor nutrition, alcohol/substance abuse and smoking [5]. The association between smoking and schizophrenia is well-established. In a recent review of the literature, the reported prevalence range of nicotine use during the prodromal phase of schizophrenia across studies was 16.6–46%. Interestingly, several studies reported an increased risk for psychosis in heavy smokers [8]. Smoking rates in patients with established schizophrenia are high, with negligible changes in smoking prevalence over time, and are associated with well-known adverse effects on the patient’s health [9]. It is therefore relevant to elucidate the habits and attitudes of patients with SMI toward smoking and plan interventions for smoking cessation accordingly.

In Greece, there are only a few studies with regard to smoking habits in patients with SMI and their views and attitudes toward smoking [10,11,12]. These studies comprise mixed patient populations, who are inpatients and outpatients in rehabilitation facilities, residing in metropolitan locations, namely, the capital of Greece, Athens. Less is known with regard to smoking habits in patients with SMI in rural or insular regions in Greece. The objective of the present study was, therefore, to explore the attitudes toward smoking in patients with schizophrenia and other SMI in residential rehabilitation facilities in insular Greece.

## 2. Materials and Methods

This is a cross-sectional, quantitative, descriptive, correlation study with closed, structured questions, based on a Likert scale. Attitudes towards smoking are measurable concepts and can be measured and presented objectively using specific questions and numerical data. The study objective is to highlight the correlation between variables, and quantitative research was most suitable in this regard.

### 2.1. The Study Setting

The present study was conducted in nine residential facilities run by the General Hospital of Corfu, Northwest Greece. A total of 153 patients are residents in those facilities. In line with international practice [13,14], patients who are eligible for such residential care suffer an SMI, mostly a psychotic or severe affective disorder, have poor psychosocial functioning, more and longer hospitalizations, worse clinical courses and outcomes; and need high levels of support. Such facilities in Greece are staffed on-site by interdisciplinary teams, which mostly comprise psychologists, social workers and nursing staff, who are recovery-oriented and enable psychosocial rehabilitation [15]. All the patients receive medication and adherence is ensured with direct supervision [16]. 

### 2.2. Recruitment/Exclusion Criteria

To participate in the study, all the individuals had to be adults, reside in a facility for psychosocial rehabilitation and have a chronic and severe mental disorder diagnosis (F20–29 and F31, respectively, according to the International Classification of Diseases, 10th revision [ICD-10]) and agree to the aims of the study. The exclusion criteria were mental retardation comorbidity, severe cognitive disturbance, not filling out the consent form, or not completing the questionnaire.

### 2.3. Participants

All 153 residents in the rehabilitation facilities were initially considered eligible for participation in the study, but 23 met the exclusion criteria (10 had co-morbid mental retardation and 13 were rated by the treating teams as severely cognitively disturbed). The remaining 130 patients were all approached for participation; 11 declined to fill out the consent form; and 16 did not return the questionnaire, despite completing the consent form. The reasons for not returning the questionnaire were not recorded, and it was hypothesized that those patients changed their initial opinion for participation in the study. Finally, a total of 103 individuals participated in the study. The current clinical status of the patients was not assessed in this study. However, psychotic decompensations or exacerbations of symptoms of mania may be disrupted for the environment of a residential facility and could lead to hospitalization. All the patients in the present sample were in a stable phase of their illness, in that they did not require hospitalization and could participate in the daily activities of the facility.

### 2.4. Procedures

The sample was recruited from psychosocial rehabilitation facilities falling under Corfu’s General Hospital administration. Permission for the study was granted by the Ethics Committee of Corfu’s General Hospital. The individuals were informed about the study procedures prior to their participation, and written informed consent was obtained from all the participants.

### 2.5. Questionnaire

The participants were asked to fill out a questionnaire consisting of statements on a five-point Likert scale created by Kourakos et al. [11], based on the semi-structured interview ‘*Smoking in a forensic psychiatric service: a survey of inpatients’ views*’ [17]. The questionnaire sought information about socio-demographic and clinical characteristics (number and length of hospitalizations, diagnosis according to ICD-10, medications, comorbidities) and the patient’s smoking habits, including the duration of smoking, age and reasons for starting smoking, number of cigarettes per day and their attitudes and opinions about smoking and health (10 items), attempts to and difficulty in quitting smoking (11 items) and smoking in residential facilities (12 items).

### 2.6. Statistical Analysis

The data analyses were performed using SPSS version 24.0. Frequency distributions (f%) were used to describe the respondents’ demographic characteristics. A factor analysis was conducted on the questionnaire items’ frequency scores to determine the number of factors. The Kaiser–Meyer–Olkin (KMO) measure of sampling adequacy was conducted to determine whether the data set was factorable. Values of at least 0.60 are required for good factor analysis [18]. The Cronbach’s alpha reliability coefficient was employed to assess the internal consistency to determine if the elements that form the questionnaire possess a sense of uniformity.

Once the factor structure was identified, the statistically significant differences in variables were evaluated employing a *t*-test, if they presented a normal distribution, and, if not, a Kruskal–Wallis test. A post hoc Bonferroni test, also called a multiple comparison test, was conducted to identify exactly which groups differ from each other. Spearman’s non-parametric correlation coefficient was employed to examine the relationship between the categorical variables or the variables that do not have a standard distribution. The Chi-square independence test was utilized to check for any dependence between the qualitative nominal variables. A *p*-value of <0.05 was considered statistically significant. *p*-value adjustments for multiple comparisons in order to reduce the probability of committing false statistical inferences were included in the present research.

## 3. Results

### 3.1. Demographic Data

The total number of participants included in this study was 103 individuals. The socio-demographic and clinical characteristics of the patients are presented in Table 1. Almost two-thirds of the participants were male, and the majority (60.2%) were 41–60 years old. The patients had been mostly raised in an urban (38.8%) or a semi-urban (26.2%) area, and more than half had only primary education. With regard to marital status, the participants were mostly unmarried (76.7%) with no children (79.4%). 

### 3.2. Medical Diagnosis, Medication, Length of Stay

The most common diagnosis in the present sample was schizophrenia (71.2%), followed by other psychotic disorders (9.6%) and bipolar disorder (8.7%). The patients’ primary medication was antipsychotics (91.3%), antidepressants (36.9%) and antiepileptics (19.4%). Almost half of the patients had been living in the rehabilitation facility for 6–10 years, whereas about a quarter of the patients had been living in the facility for 5 years or less.

### 3.3. Questionnaire Items in the Sample: Number of Factors and Psychometric Properties

The 3-factor structure, which was found to represent the data most accurately, accounted for 54.12% of the explained variance. Factor 1 accounts for 25.44% of the overall variability and encompasses four of the five questions related to the “difficulty in quitting smoking” aspect. One question in the “difficulty in quitting smoking” factor was excluded because it created reliability issues. Factor 2 accounts for 16.33% of the total variance and encompasses all questions regarding “avoiding smoking in residential facilities” and one question regarding “difficulty in quitting smoking.” Factor 3 accounts for 12.35% of the total variability and includes all questions related to “confidence in a non-smoking reference person.” The factor analysis was deemed successful, as 92.30% of the 13 questions in the questionnaire, or 12 out of 13, were properly allocated. 

The internal consistency of the questionnaire was calculated using Cronbach’s alpha reliability coefficient. The value of Cronbach’s alpha is equal to 0.611 (α = 0.611), indicating a good level of consistency between the items and the reliability of the questionnaire. The 3 factors also show a good degree of internal consistency (Factor 1: α = 0.668, Factor 2: α = 0.754, Factor 3: α = 0.926). 

### 3.4. Smoking Profile, Habits and Attitudes of the Patients

The majority of the patients (n = 90, 87.4%) reported having smoked in the past, and, from those, 78.8% (n = 71, 68.9% of the total sample) are current regular smokers, having started at the age of 21.6 (SD = 8.2) years, been smoking for 29.3 (SD = 12.2) years and consuming 18.5 (SD = 13.8) cigarettes per day, mostly (85.9%) filtered cigarettes. The results of the Spearman correlations found statistically significant correlations between the age and current smoking (r = −0.239, *p* < 0.05) and also between the age and the years of smoking (r = 0.717, *p* < 0.01). Employing a Kruskal–Wallis Test, a statistically significant difference was found between the years of smoking and the educational level (H(3) = 10.156, *p* = 0.017, *p* < 0.05). A post hoc Bonferroni analysis showed that the primary education-only patients had smoked for more years compared to the patients with secondary education (*p* < 0.05, adj sig = 0.044). The years of smoking showed a statistically significant difference with regards to the antidepressant medication; an independent sample *t*-test showed that the patients who were not on antidepressant therapy reported more smoking years [t(65) = 2.255, *p* = 0.027 < 0.05]. Finally, a statistically significant relationship was found between antiepileptics and current smoking; all the patients who were on anti-epileptic medication were currently smoking (x^2^ = 6.021, *p* < 0.01).

Regarding the reasons for starting smoking, an equal number of participants (33.8%) considered friends and stress or personal issues as factors that influenced the onset of their smoking habit, whereas 23.9% of the smoking patients stated that they began smoking out of curiosity. Slightly less than half of the patients (45.6%) believe that the smoking habit causes severe harm to overall health, compared to the 23.5% who have no concerns regarding the effect of smoking on health. Half of the smoking patients reported that physicians had advised them to quit smoking.

### 3.5. Difficulty in Quitting Smoking

A percentage of 64.8% of the 71 smoking patients reported having tried to quit smoking, whereas the remaining 35.2% had never tried. Only slightly more than half (50.7%) of the smoking patients reported having been advised by a physician to quit smoking. According to their beliefs on the 5-point Likert scale (1 = totally disagree to 5 = totally agree) of the questionnaire, the patients agreed that it is difficult to quit smoking (M = 4.24, SD = 1.21). Neutral views were expressed about the difficulty in quitting smoking when they see other patients smoke (M = 3.37, SD = 1.02) and when the atmosphere of the room is full of smoke (M = 3.21, SD = 1.01) and whether they had enough information about smoking cessation or not (M = 3.07, SD = 1.05).

For the majority (70.4%), there were no other difficulties in the decision to quit smoking. For the rest, who answered ‘yes’ to the question ‘Is there anything else that prevents you from quitting smoking?’, a percentage of 23.8% provided family issues, social circumstances and stress as obstacles to quitting smoking, whereas unemployment, addiction and the facility’s conditions were also reported. Most of the smoking patients (57.7%) thought they would need help to quit smoking, whereas the rest believed that, with personal will and perseverance, they would achieve quitting smoking, should they decide to do so (Table 2). Difficulty in quitting smoking was statistically significantly related to the place of birth, according to a Kruskal–Wallis Test (H(2) = 6.271, *p* < 0.05). A post hoc Bonferroni analysis showed that the patients who were raised in semi-urban regions had much more difficulty in quitting compared to urban (*p* < 0.05, adj sig = 0.095) and rural (*p* < 0.05, adj sig = 0.062) areas.

### 3.6. Smoking Habits in Psychosocial Rehabilitation Facilities

Smoking avoidance reflects gender differences in smoking habits in the facility. Female patients tend to avoid smoking in the facility more than male patients [t(101) = −2.73, *p* = 0.007, *p* < 0.05]. In addition, the patients on mood stabilizers prefer not to smoke in the rehabilitation facilities as much as patients on other medications [t(102) = 3.128, *p* = *0*.002, *p* < 0.05]. Regarding the personnel’s smoking habits in psychosocial rehabilitation facilities, the majority of patients (70.9%) had seen the staff smoking at work. On several occasions, the patients were aware of staff members smoking in the relatives’ lounge within the facility (Table 3).

The participants’ attitudes toward smoking in the facility environment were also inquired about (Table 4). They generally agreed on the rules for smoking and thought that the staff should avoid smoking in order to be a nice role model for patients and also encourage smoking cessation and smoking restriction. The patients were not sure about whether the staff or the visitors should be allowed to smoke with the patients, but they partly agreed that staff should be allowed to smoke at work. With regard to their reference person among the staff members, the patients were neutral about whether they would trust more and collaborate better with a reference person who did smoke than with someone who did not.

A Spearman’s correlation of the length of stay in the psychosocial rehabilitation facilities with variables about the habits and attitudes of the patients found statistically significant results, as presented in Table 5. In specific, a longer stay period in the facilities correlates with current smoking (r = 0.375, *p* < 0.01), efforts to quit smoking (r = 0.317, *p* < 0.01) increased belief that smoking causes harm to overall health (r = 0.257, *p* < 0.05), advice from a physician to quit (r = 0.328, *p* < 0.01) and the belief that smoking should be avoided in psychosocial rehabilitation facilities (r = 0.365, *p* < 0.01).

## 4. Discussion

To the best of our knowledge, this is the first study that explores the attitudes toward smoking in patients with SMI exclusively in rehabilitation residential facilities in insular Greece. Almost 70% of patients were current regular smokers. This rate differs from the recently reported rate of smoking (54.5%) in rural patients with schizophrenia spectrum disorders who were attending a community mental health service [19]. It would be interesting to study and elucidate the factors that affect smoking habits in community-dwelling outpatients, compared to those residing in rehabilitation facilities. According to the results of the study, most of the patients currently smoked, and even more had smoked in the past. The participants had been smoking for almost three decades, starting in their early twenties, and their daily consumption was rather high. The majority had tried to quit smoking in the past, although only half of them had been advised by a physician to quit. These findings are in line with previous research among 356 psychiatric patients (inpatients and residents in rehabilitation facilities) in Athens, Greece [11] and highlight the extent of the problem of smoking in rehabilitation facilities.

The aforementioned findings should be commented on, taking into account the Greek cultural context. According to recent data from the European Commission, Greece has the second-highest smoking prevalence in the European Union [20]. More recent research suggested that the smoking prevalence was up to 33.5% in a representative sample of adults and found current smoking to be correlated with chronic stress, depressive symptomatology, sleep problems and financial difficulties [21]. Most importantly, high rates of smoking in Greek adolescents have been reported and are strongly associated with the parents’ smoking status [22]. Moreover, as many as 72.9% of Greek adults were exposed to passive smoke, which continues to be a significant public health concern, despite consecutive anti-smoking legislation [21].

The participants in the present study considered severe stress or personal issues and their friends’ smoking habits as factors that influenced the onset of their smoking habits. Previous research has suggested that the most common cause for starting smoking among psychiatric patients was the amelioration of their symptoms [23]. Other research has highlighted the barriers to smoking cessation that are related to managing mental health issues, such as preventing relapse, controlling side effects and negative symptoms, managing anxiety, anger, irritability and sadness, and improving cognitive, motivational and problem-solving factors [24]. The patients in the present study seemed to agree on the difficulty of quitting smoking. Some of the perceived reasons were family issues, social circumstances, stress, unemployment, addiction and everyday life in the facility. Recent research has shown that several psychiatric patients linked their cigarette intake to the institutional environment, claiming that in community-based residential facilities, the majority of people smoked and felt that they needed something to do [25]. Smoking seems to have a crucial role in psychiatric patients’ everyday lifestyle and identity by helping them maintain a routine and have an activity [24]. However, the relationship between smoking and SMI may be more complex. Recent research has shown that smoking may be associated with earlier onset of bipolar disorder, higher prevalence of suicide attempts, other substance use disorders, more frequent hospitalizations, more symptoms, poor functioning and a worse prognosis, along with the well-known effects on physical health [26]. Moreover, recent Mendelian randomization studies have suggested that there is a causal relationship between schizophrenia and smoking, probably mediated by a shared genetic predisposition. That is, not only was a genetic liability for schizophrenia causally associated with a higher risk of lifetime smoking but, also, a genetic predisposition to lifetime smoking was causally associated with a higher schizophrenia risk [27,28]. Several potential biological mechanisms for the bi-directional causal effects of smoking and schizophrenia have been proposed [29]. Notably, tobacco smoking has been linked to neuropsychiatric disease through oxidative stress and neuroinflammation [30]. More specifically, smoking has been associated with a pro-inflammatory status in the brain, which has been implicated in the pathophysiology of schizophrenia [31,32] and bipolar disorder [33], at least for some patients. 

According to the results of the present study, if patients ever decided to quit smoking, they feel they would need help. Similarly, in Kourakos et al.’s research [11], when patients were asked about quitting smoking, 90% of them stated that they would need support. Another study in community mental health centers reported that, from a total of 44% of the smokers who were interested in smoking cessation treatment and 25% who would like to receive smoking cessation counseling treatment, only 13% were currently using such medication and 5.4% receiving consultation [34]. These findings indicate that more patients are interested in smoking cessation treatment than are actually receiving it [34]. Both in the present study and in Kourakos et al.’s study [11], approximately only half of the patients were advised by a physician to quit. The lack of guidance for smoking cessation can be explained by the results of previous research in which 91% of the participants’ psychiatrists reported “patients not interested” as a barrier that limited their smoking cessation treatment practice [34]. This finding is in line with the Himelhoch et al. study [35], in which 77% of the clinicians participating in the study believed that patients were not interested in quitting smoking. With regard to mental healthcare practitioners’ practices, it has been shown that community mental health practitioners, as well as practitioners who smoke, were less likely to apply smoking cessation practices to patients [36]. In another study reviewing mental health professionals’ attitudes toward smoking and smoking cessation among people with mental illnesses, the most common recorded beliefs were that patients are not interested in quitting, quitting is too much for patients to handle, and smoking is perceived as “the norm” by many practitioners. Other barriers reported by practitioners were lack of time, confidence and training [37]. Accordingly, little information about the effects of smoking on mental and physical health may be provided to patients. For instance, in a sample of young people attending a mental health center, although 75% acknowledged they should quit smoking in the future, most of them lacked information about the influence of smoking on mental and physical health [38]. 

Regarding attitudes towards smoking, although most patients see the staff smoking at work, they agree on the rules for smoking, and they believe that the staff should avoid smoking in order to be a good role model for patients and encourage smoking cessation and smoking restriction. This finding is in line with previous research, which found both staff and patients support a smoke-free policy rather than the continuation of smoking in psychiatric units, whereas 63% of patients perceived the smoking ban policy as a feasible option [39]. 

The majority of smoking patients in the present study had been diagnosed with schizophrenia or other psychotic disorders and reported having started smoking in their early twenties. In a study on the profile of cigarette smokers and schizophrenia [40], the mean age for tobacco use onset in patients with schizophrenia was 17.2 years old. Similar to other studies, the early age of starting smoking was positively correlated with smoking in schizophrenia [41]. Starting smoking at such an early age may indicate a genetic vulnerability [42] or a specific association between tobacco use and schizophrenia [43,44]. de Leon et al. [45] found smoking in nine out of ten cases preceding the onset of schizophrenia and indicating a possible role of tobacco smoking on the onset or perhaps the maintenance of the symptoms. According to Diaz et al. [42], schizophrenia patients had a significantly higher risk of becoming daily smokers than controls of the same age, while the analyses did not present significant differences between patients with mood disorders and the controls.

In the present study, the patients receiving anti-epileptics reported more smoking years and had higher smoking rates compared to those who were on antidepressant medication. In a previous U.S. study [46] in patients with schizophrenia living in nursing homes, it was found that an increase in depression was associated with more smoking and that depression may be related to smoking behavior. Indeed, patients on antidepressants may currently have fewer depressive symptoms than those not receiving antidepressants. However, depressive symptomatology was not assessed in the present study. 

In the present sample of patients, age was associated with smoking habits, that is, as patients grew older, they reported higher rates of non-smoking over the current period, while reporting more years of total smoking. This could be associated with the physical morbidity of older patients, although inquiring about co-morbidities was beyond the scope of this study. According to Mallet et al. [40], less tobacco use in schizophrenia is associated with negative symptoms, anticholinergic agents and clozapine or aripiprazole administration. The association between smoking and anticholinergics or specific antipsychotic medication needs further research and is not addressed in the present study. However, it should be noted that heavy smoking has been shown to reduce significantly the blood concentrations of certain drugs, such as olanzapine and clozapine, by inducing the activity of CYP1A2 [47]. 

Concerning education, the patients with primary education only reported smoking for more years compared to the higher education patients. Consistent with the literature, higher education in smoking patients with schizophrenia was associated with a lower frequency of tobacco smoking [40]. An earlier Australian study [48], which investigated the relationship between smoking and demographic characteristics, found that there was a correlation between educational level and smoking, perhaps due to the greater awareness of the harmful effects in higher education patients. In the present study, patients born in semi-urban areas had greater difficulty in quitting smoking. The literature does not provide further evidence for this finding. So, further research about smoking and the place of birth would provide interesting information. 

Concerning the duration of stay in a rehabilitation residential facility, the patients with more years of residence reported current smoking, preferring filter cigarettes and being advised by physicians to quit smoking, which they have tried to do. In addition, they agreed, to a greater extent, that smoking is harmful to their health and that it should be avoided in psychosocial rehabilitation structures. The same group of patients claimed that staff members smoke at work and, especially, in the courtyard or smoking room. In Dimopoulos’ study [49], the overall support of the patients within a residential facility is associated with greater support for smoking cessation, as well as with medication protocols that allow health professionals to choose methods that satisfy both the medical needs as well as the personal preferences of people with mental illness. In another study [41], older patients or patients with less financial comfort show a greater desire to quit smoking, related to the time spent in the facility or the time receiving treatment.

### Limitations and Potential Implications for Rehabilitation

The present study has some limitations. The sample size did not allow the application of parametric controls, which are more reliable and have statistically greater power. Future multi-center research is warranted, with stratified sampling, using a larger sample size proportional to the size of the population. In this way, it could be checked if the results of the present study are generalizable. Apart from the patients with schizophrenia, the present study does not include sub-analyses for the remaining diagnoses, e.g., bipolar disorder, as the sample representing this diagnostic category was small (n = 9). Even though smoking patterns may differ across diagnoses, such differences could not be assessed. Another limitation of the study is the missing information from a proportion of patients (n = 16) who initially agreed to participate but did not return the questionnaires. Moreover, the data collection was made through self-reporting questionnaires and may be subject to reporting bias. Finally, the current clinical status of patients was not assessed in this study; rather, all the participants were thought to be in a stable phase of their illness.

On the other hand, the results of the present study are relevant and informative for the care of chronic patients with SMI in residential facilities. The results draw attention to the need for interventions for smoking cessation in those settings. The personnel in rehabilitation facilities should not adopt a priori the belief that patients with SMI are not interested in quitting smoking but rather inquire about their attitudes and encourage patients to make efforts for cessation or to seek appropriate consultation. Moreover, mental health professionals should avoid smoking in the facilities and encourage patients to conform to the facilities’ smoking regulations. Patients with physical morbidity, especially those suffering from cardiovascular disease, should be particularly encouraged to quit smoking or referred to specialists, and, in such cases, the personnel should assume a more active role to help patients, in order to prevent premature mortality. In a previous review of the literature, several barriers to smoking cessation in patients with schizophrenia were recorded, including craving and addiction, but also the perceived increased risk of negative effects associated with quitting smoking, stress management and the maintenance of social relationships. The most consistently mentioned facilitator to quitting smoking was physical health concerns [50].

## 5. Conclusions

The results of the present study point to the importance of more holistic care of psychiatric patients and highlight the notion that this population receives substantially lower levels of care for physical disease. Smoking and smoking-related health issues are common in people with severe mental disorders. This study drew attention to smoking in residential facilities, where it appears that most patients are chronic smokers, but a large proportion recognizes the effects of smoking on health and has made efforts to quit smoking. Indeed, there is some very recent evidence that, although patients with SMI frequently engage in modifiable health-risk behaviors, most perceive the maintenance of good health as important. Accordingly, those patients could be motivated to make the necessary behavioral changes to preserve or improve health [51]. Consequently, interventions designed to educate patients with SMI regarding the importance of health and health behaviors may motivate patients to reduce their engagement in health-risk behaviors [51]. This may mean that, with regular guidance, patients may conform to a healthier lifestyle, including smoking reduction or cessation. Patients need more support and encouragement in this respect, and mental health staff in rehabilitation facilities should assume a proactive role in helping patients to receive appropriate consultation, particularly those already suffering from cardiovascular disease. There is a need for further study concerning smoking in chronic patients residing in psychosocial facilities. Targeted smoking cessation programs are especially necessary for this population group. It may be useful to take into consideration the patients’ attitudes when implementing programs for smoking cessation and to individualize interventions according to their views and preferences. Interventions regarding the facility environment and changes in smoking policies and regulations should be also considered.

## Figures and Tables

**Table 1:** Demographic and clinical characteristics of participants.

		n	%
Gender	Male	65	63.1
	Female	38	36.9
Age	21–30	9	8.7
	31–40	6	5.8
	41–50	27	26.2
	51–60	35	34.0
	61–70	22	21.4
	71–80	4	3.8
Place of birth	Rural	36	35.0
	Semi-urban	27	26.2
	Urban	40	38.8
Marital status	Unmarried	79	76.7
	Married	6	5.8
	Widow	2	1.9
	Divorced	16	15.6
Education	Primary (0–9 years)	56	54.4
	Secondary (9–12 years)	32	31.0
	Tertiary	15	14.6
Diagnosis	Schizophrenia	74	71.8
	Bipolar disorder	9	8.7
	Schizoaffective disorder	7	6.8
	Psychotic disorders	10	9.7
	Other	3	2.9
Medication	Antipsychotics	94	91.3
	Antidepressants	38	36.9
	Mood stabilizers	8	7.8
	Antiepileptics	20	19.4
Length of stay in the facility (years)	1–5	24	23.3
	6–10	51	49.5
	11–15	17	16.5
	16–20	11	10.7

**Table 2:** Smoking patients’ accounts on efforts to quit smoking.

		n	%
Is there anything else that prevents you from quitting smoking?	Yes	21	29.6
	No	50	70.4
If so, what?	Unemployment	2	
	Family issues	5	
	Addiction	2	
	Facility’s conditions	2	
	Stress	5	
	Social circumstances	5	
Trying to quit smoking, do you think you will need help to quit?	Yes	41	57.7
	No	30	42.3
If so, what would be more helpful?	Nicotine substitutes–mastic stickers	24	
	Smoking cessation sessions	6	
	Support and counseling	7	
	Other (friends, family, myself)	4	
If not, why do you believe you don’t need help? How will this be achieved?	Personal will and perseverance	23	
	Not specified	7	

**Table 3:** Patients’ accounts on staff’s smoking habits in the facility.

Dependent Variable		n	%
Does the staff smoke at work?	Yes	73	70.9
	No	30	29.1
Place where staff smokes	Smoking lounge	33	45.2
	Offices	2	2.7
	Outside	50	68.5

**Table 4:** Patients’ attitudes toward smoking in the facility environment.

Dependent Variable	Mean	SD
The facility’s smoking rules are on target	4.33	0.67
The staff should set an example and avoid smoking	3.73	0.83
The staff should encourage smokers to reduce or quit smoking	3.71	0.91
The staff should be allowed to smoke at work	3.11	0.98
Visitors should be allowed to smoke with the patients	2.99	1.01
Staff should be allowed to smoke with the patients	2.61	0.91
Trust a reference person that does not smoke more than a smoker	3.12	0.88
Cooperate better with a smoker reference person than with a non-smoker	2.83	0.96

**Table 5:** Correlation of smoking habits with patients’ length of stay in the facility.

Dependent Variables	Length of Stay in Psychosocial Rehabilitation Facilities
Current smoking	0.375
Efforts to quit smoking	0.317
Indications from a physician to quit	0.328
Beliefs of smoking being harmful to health	0.257
Smoking avoidance in psychosocial rehabilitation facilities	0.365

## Data Availability

The data are unavailable due to privacy or ethical restrictions.
